# Dual Targeting of Insulin and Venus Kinase Receptors of *Schistosoma mansoni* for Novel Anti-schistosome Therapy

**DOI:** 10.1371/journal.pntd.0002226

**Published:** 2013-05-16

**Authors:** Mathieu Vanderstraete, Nadège Gouignard, Katia Cailliau, Marion Morel, Julien Lancelot, Jean-François Bodart, Colette Dissous

**Affiliations:** 1 Center for Infection and Immunity of Lille, Inserm U1019, CNRS-UMR 8204, University Lille Nord de France, Institut Pasteur de Lille, Lille, France; 2 EA 4479, IFR 147, Universite Lille 1 Sciences et Technologies, Villeneuve d'Ascq, France; Rush University Medical Center, United States of America

## Abstract

**Background:**

Chemotherapy of schistosomiasis relies on a single drug, Praziquantel (PZQ) and mass-use of this compound has led to emergence of resistant strains of *Schistosoma mansoni*, therefore pointing out the necessity to find alternative drugs. Through their essential functions in development and metabolism, receptor tyrosine kinases (RTK) could represent valuable drug targets for novel anti-schistosome chemotherapies. Taking advantage of the similarity between the catalytic domains of *S. mansoni* insulin receptors (SmIR1 and SmIR2) and Venus Kinase Receptors (SmVKR1 and SmVKR2), we studied the possibility to fight schistosomes by targeting simultaneously the four receptors with a single drug.

**Methodology/Principal Findings:**

Several commercial RTK inhibitors were tested for their potential to inhibit the kinase activities of SmIR1, SmIR2, SmVKR1 and SmVKR2 intracellular domains (ICD) expressed in *Xenopus* oocytes. We measured the inhibitory effect of chemicals on meiosis resumption induced by the active ICD of the schistosome kinases in oocytes. The IR inhibitor, tyrphostin AG1024, was the most potent inhibitory compound towards SmIR and SmVKR kinases. *In vitro* studies then allowed us to show that AG1024 affected the viability of both schistosomula and adult worms of *S. mansoni*. At micromolar doses, AG1024 induced apoptosis and caused schistosomula death in a dose-dependent manner. In adult worms, AG1024 provoked alterations of reproductive organs, as observed by confocal laser scanner microscopy. With 5 µM AG1024, parasites were no more feeding and laying eggs, and they died within 48 h with 10 µM.

**Conclusion/Significance:**

IRs and VKRs are essential in *S. mansoni* for key biological processes including glucose uptake, metabolism and reproduction. Our results demonstrate that inhibiting the kinase potential and function of these receptors by a single chemical compound AG1024 at low concentrations, leads to death of schistosomula and adult worms. Thus, AG1024 represents a valuable hit compound for further design of anti-kinase drugs applicable to anti-schistosome chemotherapy.

## Author Summary

Schistosomiasis is a chronic, debilitating disease that affects over 200 million people in the world. The pathology of schistosomiasis is caused mainly by host immune responses to parasite eggs and due to the formation of granulomas in liver and other tissues. There is no vaccine for schistosomiasis and treatment relies essentially on a single drug, Praziquantel. However, reduced susceptibility of schistosome isolates to Praziquantel has been reported, raising serious concerns about the need to develop new drugs against schistosomes. Receptor tyrosine kinases (RTKs) control many cellular and developmental processes and they are important targets in cancer therapy. In this paper, we have investigated the possibility to fight schistosomes by targeting with a single drug, insulin receptors (IRs) involved in parasite growth and metabolism and Venus Kinase Receptors (VKRs) which are unusual IR-like RTKs expressed in the parasite reproductive organs of *Schistosoma mansoni*. Diverse RTK inhibitors have been tested on kinase activities of these RTKs. The well-known IR inhibitor, tyrphostin AG1024, was demonstrated to be a potent inhibitor of both *S. mansoni* VKRs and IRs, able to induce *in vitro* death of larvae and adult worms at micromolar doses. AG1024 could represent a good hit compound for the development of novel drugs against schistosomes.

## Introduction

Schistosomiasis is the second important parasitic disease in the world. This water-borne disease occurs in over 70 tropical and subtropical countries, mainly in sub-Saharan Africa, with 200 million individuals infected, and a number of deaths estimated to be more than 200 thousands annually [1], [2]. Diverse programs aimed at a reduction of parasite transmission, including the control of vector snail populations or the improvement of sanitation conditions and water supplies, but mass treatment of human populations by chemotherapy remains the most efficient way to combat schistosomiasis [3]. Treatment relies essentially on the use of Praziquantel (PZQ), a safe and affordable drug effective against the three major human schistosome species and recommended by WHO to reduce morbidity and mortality caused by this disease. However, massive administration of PZQ in endemic areas, and the necessity to reiterate treatments because of the ineffectiveness of the drug towards immature parasites, have raised serious concerns regarding the development of parasite resistance to PZQ [4]. Therefore, intensive efforts have been made in recent years to identify novel schistosome molecular targets for chemotherapy [5] and protein Tyrosine Kinases (TKs) have been considered as good candidates because of their essential roles in development and metabolism [6]–[8]. Receptor tyrosine kinases (RTKs) regulate many cellular activities such as proliferation, migration or differentiation, and they are the major TK signalling protagonists, being able to integrate perception, response to extracellular signals and propagation by phosphorylation of intracellular targets [9]. Cancers are often associated with deregulation of RTK activity, and these receptors, such as Epidermal Growth Factor receptor HER-2, c-Kit or VEGF-R, constitute pertinent chemotherapeutical targets in diverse anti-cancer therapies [10]–[12]. Insulin-like Growth Factor 1 (IGF-1) receptor is also commonly overexpressed in cancer and its activation affects cell proliferation, adhesion, migration and cell death [13]. Blocking IGF-1R prevents tumor cell growth and increases apoptosis in malignant cells [14]. Moreover, insulin receptor (IR), closely related to IGF-1R, is also overexpressed in many cancers. Its activation has been shown to compensate IGF-1R inhibition in malignant cells, thus validating the interest of co-targeting IGF-1R and IR in cancer [15].

IR/IGFR molecules are conserved in a large variety of eumetazoan species, from sponges to mammals [16]. Two receptors of the IR family, SmIR1 and SmIR2, have been characterized in *Schistosoma mansoni*, and display differences in their tissue localization. SmIR-1 is expressed in muscles, intestinal epithelial cells and at the basal membrane of the tegument [17], colocalized with SGTP1 and SGTP4 schistosome glucose transporters [18]. SmIR2 is massively expressed in parenchymal cells of adult schistosomes, suggesting that the two receptors could have distinct functions [17]. Two IR members have been also found in *Schistosoma japonicum* (SjIR1, SjIR2) which are highly similar to SmIR1 and SmIR2 respectively [19]. In both schistosome species, these receptors might have conserved IR function in the regulation of glucose uptake, since treatment by IR specific inhibitors affect significantly glucose entry in parasites [19], [20].

Two additional RTKs (SmVKR1 and SmVKR2) with intracellular kinase domains similar to that of SmIRs were also characterised in *S. mansoni*. They were named VKR for Venus Kinase Receptor since they contain in their extracellular part an atypical Venus FlyTrap (VFT) motif usually found in G-protein-coupled receptors of class C. SmVKRs are members of a novel family of RTKs discovered few years ago [21], present only in invertebrates and activable by amino-acids [22], [23]. These receptors are highly expressed in larval stages of the parasite as well as in ovaries of female worms, suggesting functions in development and reproduction [23]. Considering the potential importance of SmIRs and SmVKRs in development, but also in metabolism and reproduction, the striking similarity observed between the catalytic domains of the four receptors led us to postulate that targeting simultaneously these four effectors by a single compound would be highly detrimental for the parasites and might possibly represent a novel multiple target strategy against schistosomes.

Here, we analyzed the potential of several IR and RTK inhibitors to inhibit kinase activities of both SmIR and SmVKR kinase domains recombinantly expressed in *Xenopus* oocytes. Among the different compounds tested, tyrphostin AG1024 emerged as the most potent inhibitor towards the four receptors. *In vitro* experiments then demonstrated that treatment with AG1024 led to dramatic effects on the viability of larval and adult schistosomes as well as on the fertility of adult worms.

## Materials and Methods

### Ethics statement

All experiments involving hamsters within this study have been performed in accordance with the European Convention for the Protection of Vertebrate Animals used for Experimental and other Scientific Purposes (ETS No 123; revised Appendix A) and have been approved by the committee for ethics in animal experimentation of the region Nord Pas de Calais France (authorisation No. AF/2009) in the local animal house of the Pasteur Institute of Lille (Agreement No. A59-35009).

### Parasite material

A Puerto-Rican strain of *S. mansoni* was maintained by passage through albino *Biomphalaria glabrata* snails and *Mesocricetus auratus* golden hamsters. Adult schistosomes were collected by portal perfusion from infected hamsters at 42–45 days p.i. Schistosomula were prepared as described previously [24].

### Molecular cloning of SmIR and SmVKR intracellular domains

Intracellular domains (ICD) of SmIR1, SmIR2, SmVKR1 and SmVKR2 were amplified by PCR from pcDNA3.1 plasmids encoding the receptor full-length sequences [17], [21], [23] using Fwd 5′-CCggatccAACGGAGAATTTCACGGAAACGTCTGCAG-3′ and Rev 5′-CCctgcagTCAAATATATAAGGAAGAAGATGTGAATG-3′ primers with BamH1 and Pst1 sites for SmIR1 ICD; Fwd 5′-CCgaattcCGTCGTTATTATTTAAAGGTTACAGCTTGG-3′ and Rev 5′-CCggatccTTATGCGATAACGTTTCTAGTTCTACTTAG-3′ primers with EcoRI and BamH1 sites for SmIR2 ICD; Fwd 5′-GGgaattcGTCAACCATATGAAAACCTTTG-3′ and Rev 5′-CCctgcagTCAAGGTAGAAACGCTAAACTGTTATC-3′ primers with EcoR1 and Pst1 sites for SmVKR1 ICD; Fwd 5′-AATggatccTAAACGGTCTTCCTACCGGAAAG-3′ and Rev 5′-CCccatggCGACGTAAACTGAAAGAAATTGAAAATCG-3′ primers with BamH1 and Nco1 sites for SmVKR2 ICD. PCR products were inserted into pCR2.1 TOPO vector (Invitrogen) before cloning in phase with the myc epitope tag in pGBKT7 expression vector (Clontech). pGBKT7 plasmids containing wild-type SmIR1^WT^, SmIR2^WT^, SmVKR1^WT^ and SmVKR2^WT^ ICDs were further submitted to site-directed mutagenesis in order to render the kinase protein domains constitutively active. For this, the second amino-acid next to the conserved YY autophosphorylation site contained in SmIR and SmVKR kinase domains was replaced by a glutamic residue according to the procedure already described [22]. Constitutively active mutants of SmIRs were obtained using 5′-CGTCTTGTAAATAATCAAGAATATTATAGAgAAATTGGACAAGC-3′ mutated sequence and its reverse complement to generate SmIR1^YYRE^ and 5′-CAGATGTTTATGGACATAATTATTATCACgAAACAAGTCATGC-3′ and reverse complement sequences for the SmIR2^YYHE^ mutant. SmVKR1^YYRE^ and SmVKR2^YYRE^ constructs were obtained as described in [23].

### Protein expression in *Xenopus laevis* oocytes

cRNA encoding wild-type or constitutively active mutants of receptor ICDs were produced using the T7 mMessage mMachine Kit (Ambion, USA). cRNAs were transcribed from T7 promoter-containing pGBKT7 plasmids (1 µg) previously linearised by HindIII restriction enzyme. cRNAs were precipitated by 2.5 M LiCl, washed in 70% ethanol, resuspended in 20 µl diethylpyrocarbonate (DEPC)-treated water, and quantified by spectrophotometry. cRNAs were analysed in a denaturating agarose gel. Gel staining with 10 µg ml^−1^ ethidium bromide allowed to confirm correct sizes and of absence of abortive transcripts. cRNA preparations (1 mg ml^−1^) were microinjected in *Xenopus* oocytes (stage VI) according to the protocol previously described [25]. Each oocyte was injected with 60 nl of cRNA in the equatorial region and incubated at 19°C in ND96 medium (96 mM NaCl, 2 mM KCl, 1 mM MgCl2, 1.8 mM CaCl2, 5 mM HEPES pH 7.4 supplemented with 50 µg/ml streptomycin/penicillin, 225 µg/ml sodium pyruvate, 30 µg/ml trypsin inhibitor) for 18 h. For inhibitor treatments, oocytes were incubated with tyrphostin AG538, AG1024, AG1478, HNMPA-(AM)3 (Santa Cruz Biotechnology), SU11274 or BIBF1120 (Selleckchem) at different concentrations. In all cases, germinal vesicle breakdown (GVBD) was detected by the appearance of a white spot at the apex of the cell, a witness of oocyte progression from G2 to M phase of the cell cycle.

### Immunoprecipitation and Western blot analyses

Immunoprecipitation of myc-tagged ICD proteins expressed in oocytes was performed according to the procedure described previously [25]. Following 18 h of cRNA injection, oocytes were lysed in buffer (50 mM HEPES pH 7.4, 500 mM NaCl, 0.05% SDS, 0.5% Triton X100, 5 mM MgCl2, 1 mg/ml bovine serum albumin, 10 µg/ml leupeptin, 10 µg/ml aprotinin, 10 µg/ml soybean trypsin inhibitor, 10 µg/ml benzamidine, 1 mM sodium vanadate) and centrifuged at 4°C for 15 min at 10,000 g. Supernatants were incubated with anti-Myc (1/100; Invitrogen) antibodies for 4 h at 4°C. Protein A-Sepharose beads (5 mg, Amersham Biosciences) were added for 1 h at 4°C. Immune complexes were collected by centrifugation, rinsed three times, resuspended in Laemmli sample buffer, and subjected to a 10% SDS-PAGE. Immune complexes were analyzed by Western blotting using anti-myc (1/50,000) or pY-20 (1∶10,000; anti-phosphotyrosine, BD Biosciences) antibodies and the advanced ECL detection system (Amersham Biosciences).

### Treatment of parasites with inhibitor

500 schistosomula were incubated for 7 days in 24-well plates containing 2 ml of M199 medium (Invitrogen) supplemented with HEPES 10 mM, pH 7,4, antibiotic/antimycotic mixture (Sigma, 1.25%) and FCS (10% Gibco) (referred as M199 complete medium) with different concentrations (from 1 to 50 µM) of tyrphostin AG1024 (Santacruz Biotechnology) dissolved in DMSO. Culture medium was refreshed daily. Parasite mortality was assessed by eye each day using three criteria: absence of motility, tegument defects and granular appearance. A minimum of 300 larvae was observed for each condition, and the ratio dead larvae/total larvae calculated in three independent experiments.

Twenty adult paired couples of *S. mansoni* were incubated at 37°C in a 5% CO_2_ atmosphere in 10 ml M199 complete medium in the presence of tyrphostin AG1024 at different concentrations (from 1 to 10 µM) for 5 days. Culture medium was refreshed daily. The number of paired couples was estimated every day by stereomicroscopy. In each well, medium containing the eggs was harvested every day, and fractions were then pooled and centrifuged. Total number of eggs was determined from three independent countings.

### TUNEL assays

Apoptosis was detected using the Terminal deoxynucleotidyl transferase dUTP nick end labeling (TUNEL) method and the In Situ Cell death detection kit (Roche). Briefly, 2,000 schistosomula were incubated for 48 h in 6-well plates containing 2 ml of M199 complete medium without or with 10 or 50 µM AG1024, then fixed in formaldehyde 2%. Labeling of schistosomula with DAPI and TMR red-dUTP was performed according to manufacturer's instructions and TUNEL-positive parasites were observed by fluorescence using an AxioImager Z1-Apotome microscope (Zeiss).

### CLSM examination

After 5 days of culture, worms were fixed for at least 24 h in AFA (ethanol 95%, formalin 3% and glacial acetic acid 2%), stained for 30 min with 2.5% hydrochloric carmine (Certistain, Merck), and destained in acidic 70% ethanol. Following dehydration in 70%, 90% and 100% ethanol, 1 min each, worms were preserved as whole-mounts in Canada balsam (Merck) on glass slides [26], [27]. The morphology of the reproductive organs of parasites was observed using a Confocal Laser Scanning Microscope (CLSM) Leica TCS SP2 microscope, with a 488 nm He/Ne laser and a 470 nm long-pass-filter under reflection mode.

## Results

### Expression of catalytically active domains of SmIRs and SmVKRs

**Figure 1:**
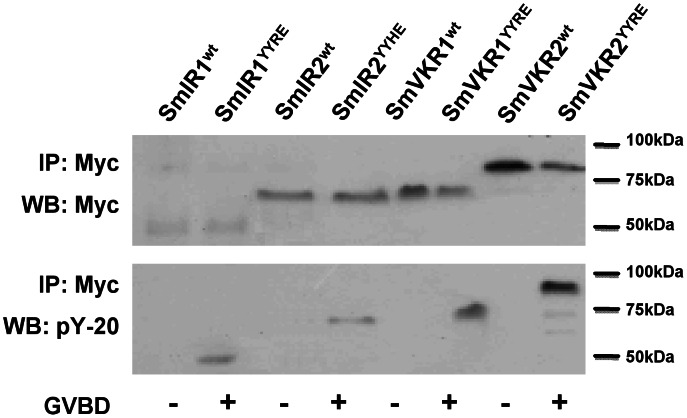
Expression of SmIR and SmVKR intracellular domains in *Xenopus* oocytes. Sets of 20 oocytes were microinjected with cRNA encoding Myc-tagged versions of SmIR1, SmIR2, SmVKR1 and SmVKR2 intracellular domains, in their native (^wt^) and constitutively active forms (^YYXE^). Immunoprecipitation and Western Blot analyses were performed using anti-Myc and pY-20 antibodies. Tyrosine phosphorylated active proteins could trigger meiosis resumption in oocytes (monitored by GVBD).

Intracellular domains (ICD) of SmIRs and SmVKRs were amplified and cloned into the pGBKT7 vector which contains the T7 promoter sequence required for *in vitro* transcription. The expression of myc-tagged proteins of SmIR and SmVKR ICDs was obtained following injection of their respective cRNAs in oocytes. Proteins could be detected by western blot analysis of oocyte lysates with anti-myc antibodies. Myc-tagged proteins were detected at molecular weight of 41 kDa for SmIR1, 69 kDa for SmIR2, 68 kDa for SmVKR1 and 81 kDa for SmVKR2 constructs (Figure 1). Several studies have demonstrated that the *Xenopus* oocyte is a suitable model for expressing *S. mansoni* proteins and particularly for studying phosphorylating activity of protein kinases [23], [25], [28]–[32]. In oocytes, which are giant cells naturally blocked in prophase I of meiosis I, the kinase activity of any exogenous recombinant kinase is able to trigger resumption of meiosis and passage into metaphase II, following germinal vesicle breakdown (GVBD), a process easily detected by the appearance of a white spot at the animal pole of the oocyte. In order to analyze receptor kinase activities, we prepared constitutively active mutants by site-directed mutagenesis. In YYxE mutants, a glutamic (E) residue was introduced near the YY autophosphorylation site, in order to mimic its phosphorylation and to induce spontaneous kinase activation.

Results shown in Figure 1 indicated that SmIR1^YYRE^, SmIR2^YYHE^, SmVKR1^YYRE^ and SmVKR1^YYRE^ mutant proteins were effectively recognized by anti-phosphotyrosine antibodies, confirming their potential to autophosphorylate and thus demonstrating their constitutive kinase activity. As expected, only the oocytes expressing constitutively active kinases, but not the wild-type ones, underwent GVBD. The number of oocytes undergoing GVBD could be used in following tests as an indicator of the kinase activity of ICD proteins.

### Inhibition of SmIR and SmVKR kinase activities by commercial inhibitory compounds

**Figure 2:**
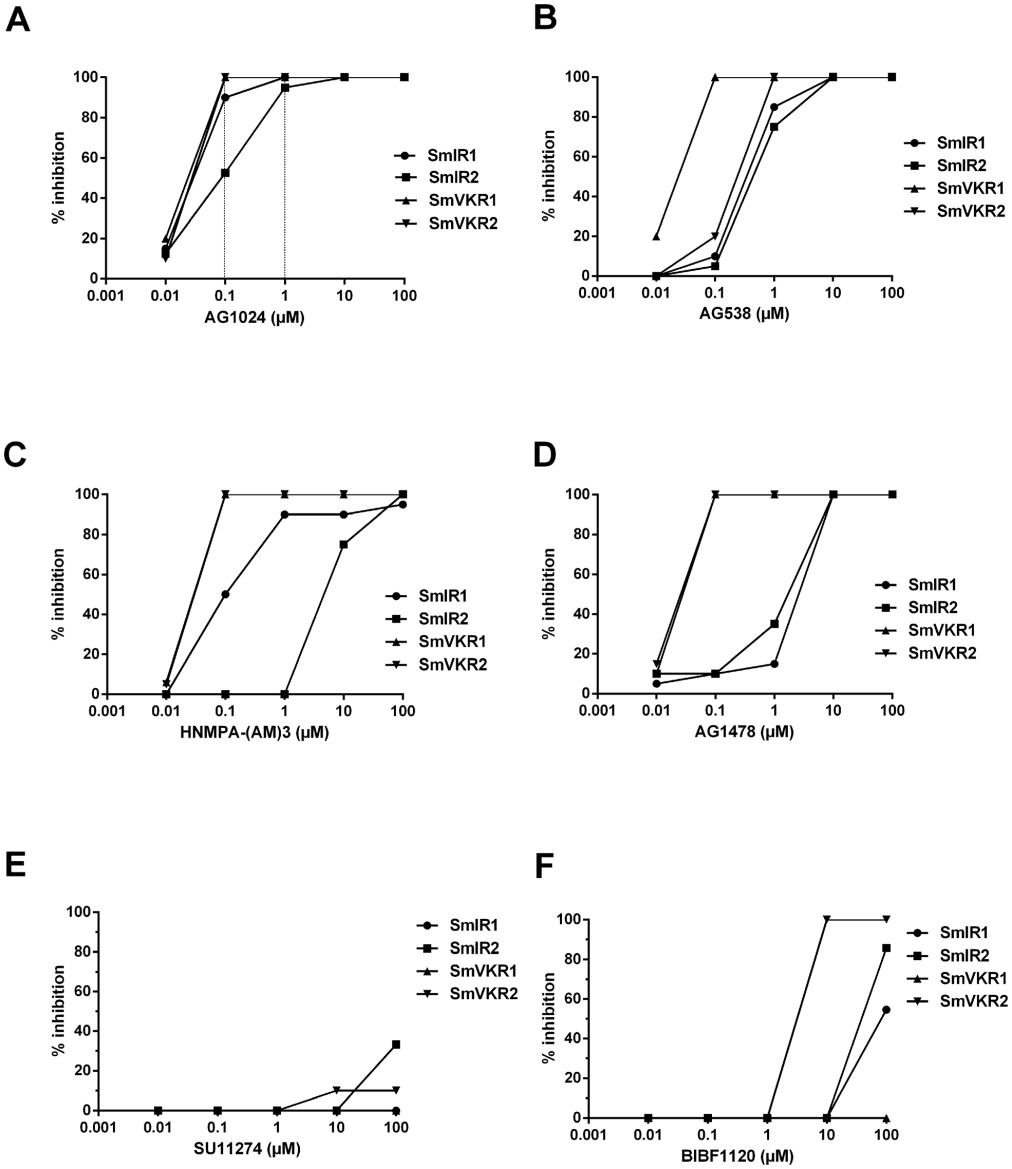
Sensitivity of SmIR and SmVKR kinase activities to various RTK inhibitors. Sets of 20 oocytes were injected with cRNA encoding constitutively active forms of SmIR and SmVKR cRNA (as described in Figure 1), and incubated with variable concentrations of tyrphostins AG1024 (A), AG538 (B), HNMPA-(AM)3 (C), AG1478 (D), SU11274 (E) and BIBF1120 (F). In each set, the percentage of oocytes that underwent GVBD was calculated. Results are expressed by inhibition in % of control oocytes and represent the mean of three independent experiments.

**Figure 3:**
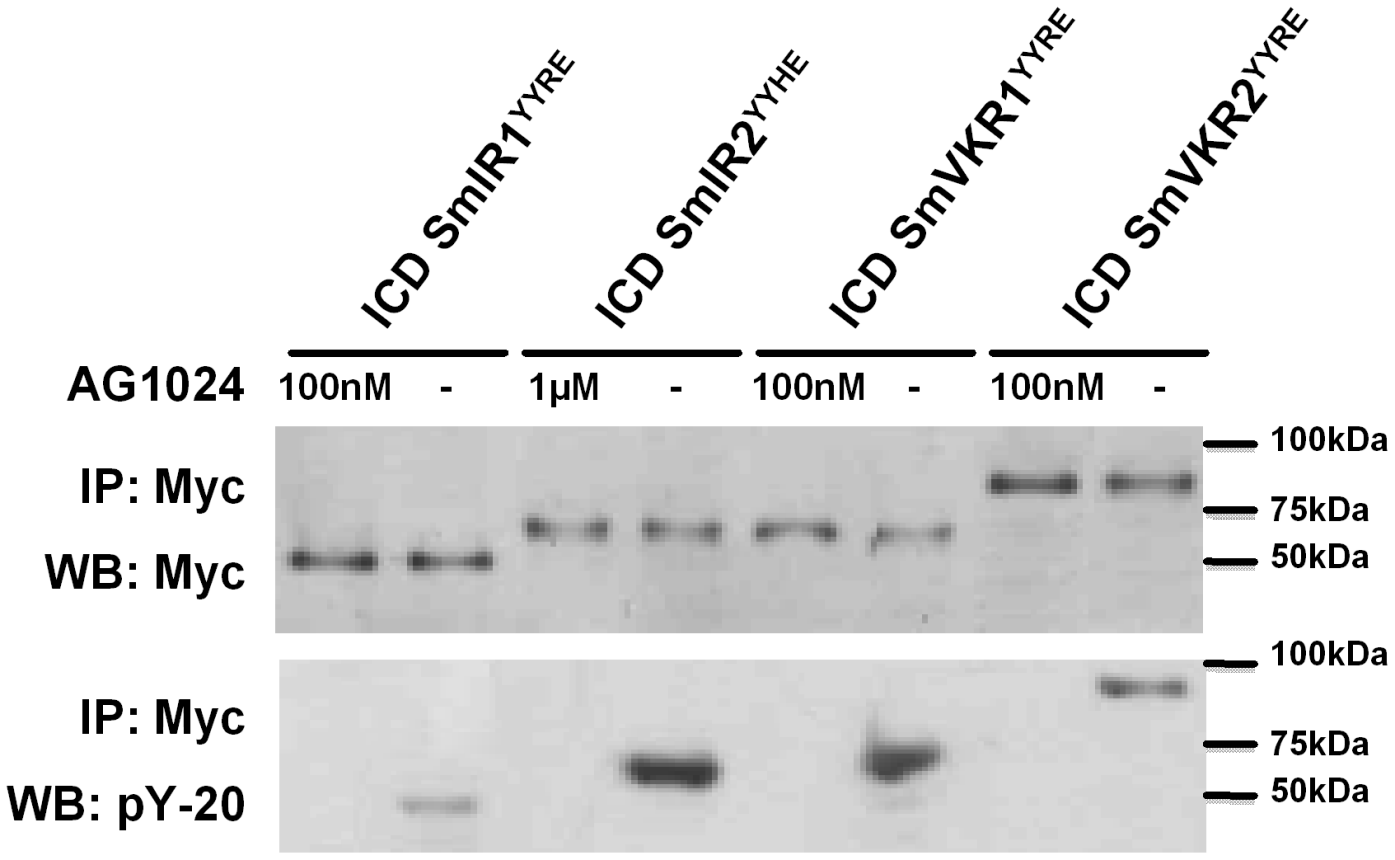
Tyrphostin AG1024 inhibits SmIR and SmVKR autophosphorylation. SmIR1^YYRE^, SmIR2^YYHE^, SmVKR1^YYRE^ and SmVKR2^YYRE^ proteins were expressed in *Xenopus* oocytes (as in Figure 1) incubated in ND96 medium containing or not the minimal concentration of AG1024 able to inhibit 100% of their ability to induce GVBD (respectively 100 nM, 1 µM, 100 nM and 100 nM). Immunoprecipitation and Western Blot analyses were performed using anti-Myc and pY-20 antibodies. Inhibition of autophosphorylation correlated with the disability of kinases to induce GVBD (see Figure 2).

SmIR1^YYRE^, SmIR2^YYHE^, SmVKR1^YYRE^ and SmVKR1^YYRE^ ICDs were expressed in *Xenopus* oocytes and we tested the capacity of several TK inhibitors to inhibit their potential to induce GVBD in oocytes. As the kinase domains of SmIR and SmVKR proteins were previously shown to be highly similar to those of insulin receptors [21]–[23], we analysed the effect of three well-known IR and/or IGFR inhibitors (tyrphostins AG538 and AG1024, HNMPA-(AM)3) on schistosome receptor ICD kinase activity. Tyrphostin AG1478 (EGFR inhibitor), SU14278 (Met receptor inhibitor) and BIBF1120 (FGFR inhibitor) were tested in parallel at different concentrations (Figure 2A). First results showed that both SmIR and SmVKR kinases were sensitive to IR/IGFR inhibitors and that among these three compounds, AG1024 was the most effective, able to inhibit at 100% GVBD in oocytes expressing SmVKR1, SmVKR2, SmIR1 at a 0.1 µM dose, and SmIR2 at a 1 µM dose. Complete inhibition of GVBD induced by ICDs was obtained with the IGFR inhibitor AG538 at 1 µM, except in SmVKR1-expressing oocytes in which 0.1 µM AG538 was sufficient to totally inhibit the activity. The effectiveness of HNMPA-(AM)3 was similar to that of AG1024 on SmVKR1 and SmVKR2 (0.1 µM) but this drug was less effective on SmIR1 and SmIR2, that required respectively minimal doses of 1 and 10 µM to be inhibited. Surprisingly, AG1478, a potent inhibitor of EGFR, was effective on SmVKR1 and SmVKR2 at low doses (0.1 µM) whereas its action on SmIR1 and SmIR2 was relatively weak (10 µM needed to inhibit 100% GVBD). As expected, the Met kinase inhibitor SU14278 had no detectable activity on SmVKRs and SmIR1 (only 40% inhibition of GVBD in SmIR2-oocytes at 100 µM) and FGFR inhibitor BIBF1120 was also inactive on the four schistosome kinases. From these data, we concluded that AG1024 was the most potent drug to inhibit both SmIRs and SmVKRs, since it blocked completely the activity of SmIR1, SmVKR1 and SmVKR2 ICDs at a 100 nM concentration, and SmIR2 kinase activity at 1 µM. Western blot results (Figure 3) confirmed that inhibition of GVBD in the presence of the drug was associated with an absence of tyrosine phosphorylation and kinase activation for each ICD.

### AG1024 induces *in vitro* schistosomula death by apoptosis

**Figure 4:**
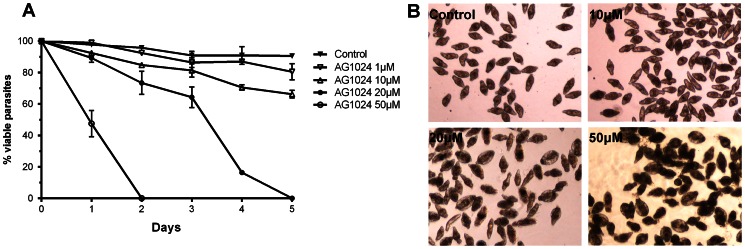
Dose-dependent effect of AG1024 on viability of cultured schistosomula of *S.mansoni*. (A) Viability of schistosomula cultured for 5 days without or with 1, 10, 20 or 50 µM of AG1024. Results are expressed as % of surviving larvae (mean ±SD, three independent experiments). (B) Morphological examination of schistosomula following 48 h incubation with 10, 20 and 50 µM AG1024. All schistosomula died within 48 h with 50 µM AG1024, characterized by their dark appearance, granular aspect and tegumental abnormalities.

In order to investigate the effect of AG1024 on the viability of *S. mansoni* larvae, 24 h-old schistosomula were cultured *in vitro* for 5 days with different concentrations of AG1024, with daily renewal of drug-containing medium. Parasite death was assessed by eye, following three criteria: loss of motility, tegument alterations and granular aspect (Figure 4B). We observed that AG1024 treatment led to parasite death in a time and dose-dependent manner. Indeed, 50 µM of AG1024 induced 100% of parasite death within 48 h, whereas five days were required with 20 µM. Treatment of schistosomula with 1 and 10 µM AG1024 much lower affected parasite viability (with 15 and 30% mortality within 5 days respectively) (Figure 4A).

**Figure 5:**
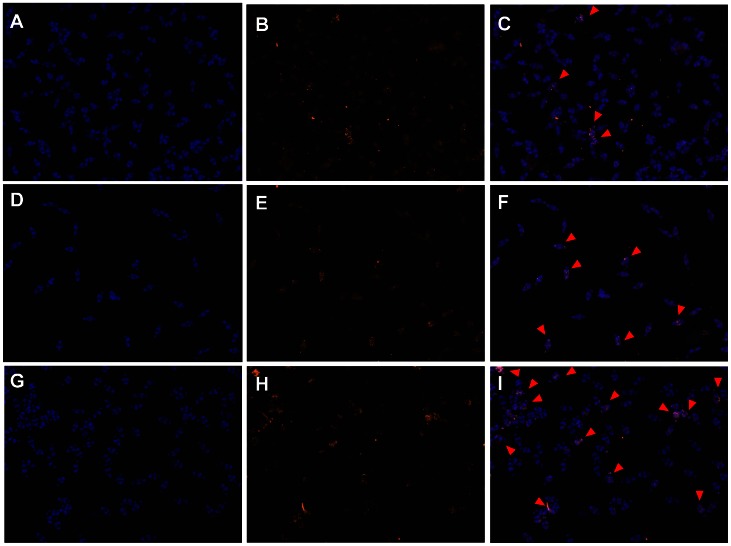
AG1024 induces apoptosis in *S.* mansoni schistosomula in culture. Schistosomula were treated for 48 h with DMSO (A, B, C), 10 µM AG1024 (D, E, F) or 50 µM AG1024 (G, H, I), then fixed and stained with DAPI and TUNEL. DAPI (A, C, F), TUNEL (B, D, G) and merged (C, E, H) pictures are shown. TUNEL-positive schistosomula are indicated by arrows. Their number increased in the presence of AG1024, reaching a maximum for the 50 µM treatment (G, H).

Since AG1024 is known to trigger apoptosis in human cell lines [33], we evaluated the occurrence of apoptosis-induced death in schistosomula using a TUNEL assay. In these experiments, schistosomula were treated with 10 or 50 µM AG1024 for 48 h, then fixed and stained with DAPI and TUNEL (Figure 5). Results indicated that AG1024 could induce apoptosis in schistosomula in a dose-dependent manner. Taken together, these results strongly suggest that AG1024 could lead to schistosomula death by inducing apoptotic signals, through inhibition of SmIR and SmVKR kinases.

### AG1024 has fatal impact on *S. mansoni* adult worms

**Figure 6:**
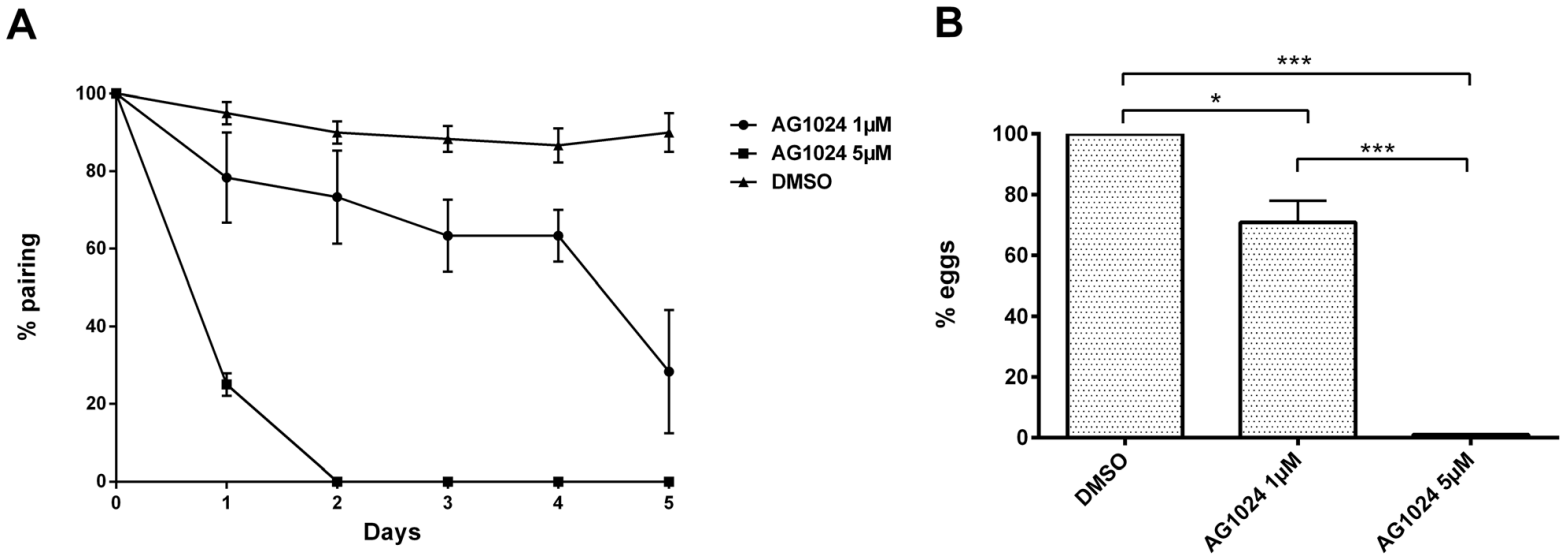
Treatment by AG1024 induces unpairing of adult worms and decrease of egg laying in a dose and time-dependent manner. Freshly perfused paired worms (20 couples) were incubated for 5 days with DMSO solvent, 1 µM or 5 µM of AG1024. (A) Percentages of paired worms were determined every 24 h. Results are expressed as the mean (± SEM) of three independent experiments. (B) In each assay, the total number of eggs laid during the 5 days was determined. Results were expressed as the percentage of eggs laid by AG1024-treated worms compared to controls (mean ± SD, three independent experiments). Statistical analyses were performed using the Student's t-test, and significance is displayed as follows: *: p<0,05, **: p<0.01, ***: p<0.001.

The effect of AG1024 was also studied on adult worms *in vitro*. In these experiments, *S. mansoni* couples were cultured with different concentrations of AG1024, and we monitored pairing behaviour and egg production for each condition during 5 days. Results showed that drug treatment had drastic effects on parasite fitness and egg production. Indeed, 1 µM AG1024 was affecting the stability of worm pairing, showing only 30% of couples still paired after 5 days (Figure 6A) and 30% decrease of egg laying (Figure 6B). Striking effects of AG1024 on schistosomes were registered at 5 µM, a dose at which worms were no more paired and egg laying almost stopped at day 2. At this time point, worms also suffered of tetany and were drifting as a consequence of their inability to stick to well bottom walls. Gut peristalsis stopped after 5 days, suggesting that SmIRs and/or SmVKRs may also regulate functions in gastrodermis and/or smooth muscles. Finally, at higher concentrations (from 10 to 50 µM), AG1024 induced adult worm death within a 2 to 5 day period (not shown).

**Figure 7:**
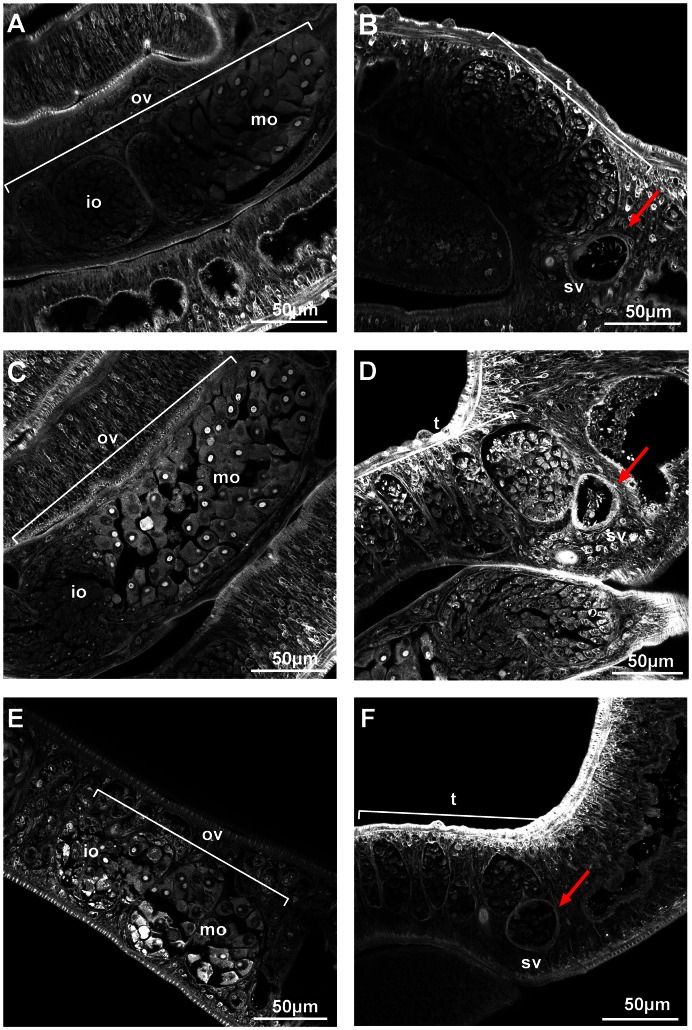
Alterations of reproductive tissues and organs under treatment by AG1024 of *S.* mansoni adult worms cultured *in vitro*. Freshly perfused paired worms (20 couples) were incubated for 5 days with DMSO solvent (A, B), 1 µM AG1024 (C, D) or 5 µM AG1024 (E, F), then fixed and stained with hydrochloric carmine. CLSM examinations showed, particularly at 5 µM, a significant size reduction of female ovary (E), associated with changes in the distribution of immature (io) and mature (mo) oocytes within the ovary lobes (ov), as compared to the control (A). In males treated with AG1024, sperm vesicle (sv, indicated by red arrows) is full of round undifferentiated germinal cells (clearly visible in F), whereas in DMSO-treated control worms it contains elongated spermatozoa. (t) testis.

**Figure 8:**
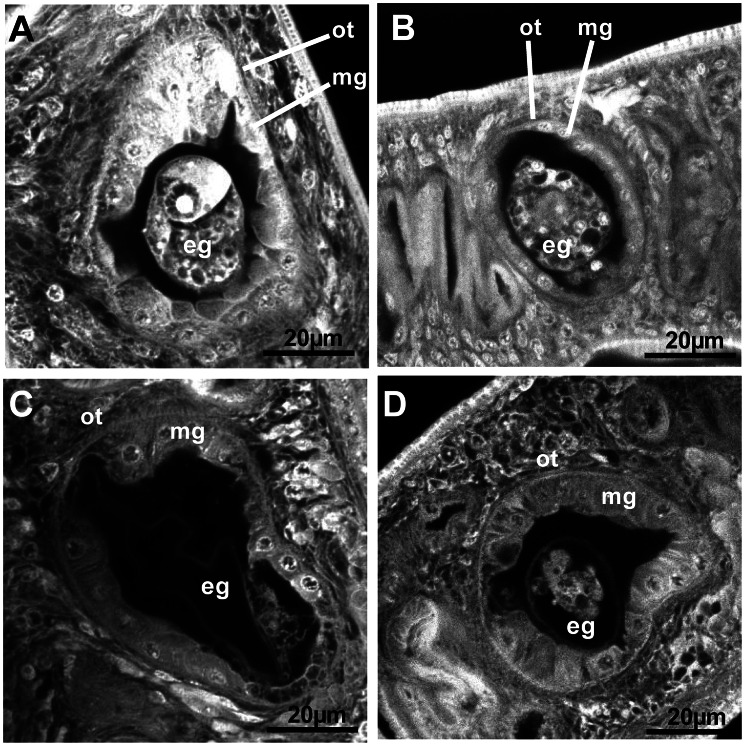
Modifications of ootype structures and egg formation in AG1024-treated *S.* mansoni adult female worms. CLSM examination centered on the female ootype of control parasites (A) or parasites treated with AG1024 1 µM (B) or 5 µM (C, D). Ot (ootype); mg (Mehlis'gland); eg (egg).

To complement these observations, we examined AG1024-treated adult worms by confocal laser scanning microscopy. Whereas no significant phenotype could be detected in gonads of adult worms treated with 1 µM of AG1024 (Figure 7 C, D), major changes occurred in worms treated with 5 µM (E, F). In females, we observed important size reduction and disorganization of the ovary, which in normal parasites (A) contains small immature oocytes in its anterior part and large mature oocytes in its posterior part. In AG1024-treated worms, immature cells were less abundant and mature cells seemed to invade the whole ovary. Focus on the ootype of female worms (Figure 8) further indicated that the drug significantly affected at 1 µM the composite structure of the egg formed in the ootype (B), inhibiting totally its formation when used at 5 µM (C and D). Moreover, we could note in treated parasites, a significant atrophy of Mehlis' glands, the cells that line the ootype [27].

In males, we could observe main changes inside of the sperm vesicle, that was full of undifferentiated cells in worms treated with 5 µM AG1024 (Figure 7 F), whereas a population of elongated spermatozoa was visible in control worms (B). These data indicated that AG1024 treatment could also affect spermatogenesis.

## Discussion

Current strategies of intervention against schistosomiasis are based on drug administration of PZQ. Since PZQ neither kills immature schistosomes nor prevents reinfection, PZQ-based control programs provide only a transitory effect on parasite transmission and a limited potential on eradication of the disease. Moreover, concerns have been raised about PZQ resistance that actually emphasize the need for new initiatives in search for alternative antischistosome compounds and in discovery of novel parasite drug targets [4], [5]. Recent studies have convincingly demonstrated the importance of protein kinases in schistosome biology and TKs have been considered as good candidates because of their essential roles in development and metabolism [6]–[8]. Targeting of Src (SmTK3) [34] and Syk (SmTK4) [31] kinases with their respective TK inhibitors herbimycin and piceatannol was shown to have a marked effect on reproduction processes of *S. mansoni* and the anti-cancer drug Imatinib (STI-571, Gleevec) that targets Abl kinases led to important alterations of parasite gastrodermis and caused the death of parasites *in vitro* [32], [35].

During the last few years we have demonstrated the peculiar nature of insulin-dependent or insulin-related signalling in schistosomes. First, two distinct IR homologs (SmIR1 and SmIR2) were found in *S. mansoni* whereas only one single IR is present in most of invertebrate species [17]. Second, two additional receptors (SmVKR1 and SmVKR2) were discovered that contain catalytic IR-like domains, and thus represent alternative candidates able to participate in IR-like pathways [21]–[23]. Such a diversification of the IR family, described for the first time in *S. mansoni* [17], was confirmed recently in *S. japonicum* and *Clonorchis sinensis* [19], [36] (Vanderstraete *et al*, submitted). This reflects a complex insulin-related network that we could consider as a real “Achille's heel” for these parasite trematodes in terms of targets for chemotherapy. Since schistosome IRs have been shown to participate in parasite metabolism by their regulatory function in glucose uptake in adult parasites [17], [19], [20] while SmVKR1 and SmVKR2 highly expressed in gonads [22], [23] play important functions in development and gametogenesis (M. Vanderstraete *et al*, to be published), we have investigated the possibility to fight parasites by dual targeting of metabolism and reproductive processes through the inhibition of their four IR-like receptors using a single drug.

In these studies, we have tested several commercial drugs known to inhibit human RTK activity and to be efficient on various cancer cells. Three inhibitors AG1024, AG538, HNMPA-(AM)3, which are specific for IR/IGFR [37]–[39] and whose detrimental effects especially on glucose uptake in adult parasites have been already described [19], [20] were analyzed along with three other compounds known to inhibit either EGFR (AG1478), or Met (SU11274) or FGF-R (BIBF1120). Inhibitory effect of these compounds was analyzed towards SmIR and SmVKR recombinant active kinases produced in *Xenopus* oocytes, a highly suitable cellular model in which we can directly relate the potential of proteins to induce meiosis resumption to their kinase activity [23], [25], [28]–[32]. Whereas tyrphostins AG1024 and AG538 were active at ≤1 µM on SmIR1 and SmIR2, surprisingly, HNMPA-(AM)3 was active only at ≥1 µM on SmIR1 and at ≥10 µM on SmIR2. The efficacy of the three IR inhibitors, was equal or even better towards SmVKRs than against SmIRs, and AG1024 emerged as the most potent drug, being able to inhibit the four receptors at a dose of ≤1 µM in the kinase assay developed in *Xenopus* oocyte. Concerning the EGFR inhibitor AG1478, its unexpected effect on SmVKRs at ≤0.1 µM was unexplained. Besides, the lower efficacy of AG1478 on SmIRs (10 µM) suggested structural differences between IR/IR-like catalytic domains of the two receptor classes. The Met receptor inhibitor SU11274 had almost no effect on SmIR and SmVKR kinases and BIBF1120 was not active at <10 µM on any of these kinases, confirming the conserved IR-like structure of SmIR and SmVKR catalytic domains.

From these data, we decided to analyze the effect of the selective inhibitor of IGF-1R, AG1024, on the viability of larval and adult stages of *S. mansoni in vitro*. We could demonstrate that AG1024 caused death of schistosomula in a dose and time-dependent manner, inducing apoptotic signals in the parasite, similarly to its effect caused on cancer cells [33]. Concerning the adult stage, results indicated that parasite couples, compared to schistosomula, were sensitive to lower amounts of the drug, and showed important loss of fitness and fertility at doses ≤5 µM. These concentrations are lower than those used on MCF7 human breast cancer cells (≥10 µM) to decrease proliferation and cause apoptosis [33], indicating the particular sensitivity of the parasites to the drug.

Since evidence has been given that gonads are important sites for the expression of SmVKR1 and SmVKR2 [22], [23], this tempts to assign in priority the decrease of egg formation and laying consecutive to AG1024 treatment, to the inhibition of SmVKR kinase activities. However, we propose that the concomitant targeting of SmIR receptors could be responsible also for alterations of reproductive processes in AG1024-treated worms. Indeed, You *et al* have shown that the insulin receptor SjIR2 was located in vitelline cells of *S.japonicum* females [19] and these authors demonstrated recently that vaccination of mice against the ligand-binding domain of SjIR2 resulted in a significant reduction of faecal eggs and liver granuloma density in infected animals, suggesting the importance of schistosome IR receptors both for nutrition (glucose consumption) and reproduction of parasites [40].

While AG1024 is a specific IGF-1R and IR inhibitor, it has been shown that AG1024 had an additional target in melanoma cells upstream of the Erk2 kinase [41]. We do not exclude that side-effects of the drug on other kinases and especially on other parasite RTKs, could contribute to the toxic effect of the drug on the parasite. Recent experiments in oocytes performed using the recombinant EGF receptor of *S. mansoni*, SER [25], demonstrated that its kinase activity was also sensitive to AG1024 at 1 µM, thus confirming the potential of this drug in the context of a multi-kinase targeting. Finally, it was shown that AG1024 was, among the tested IR inhibitors, the most toxic one for schistosomes. Tyrphostin AG538 and HNMPA-(AM) 3 had no visible effect on parasite viability *in vitro* when used at 10 µM during a 5 day culture (results not shown). Considering the large identities that exist between the kinase domains of VKR1 and VKR2 receptors from *S.mansoni* and *Schistosoma haematobium* (98% and 99% respectively) as well as between those of the IR1 and IR2 molecules of *S. mansoni* and *S. japonicum* (74% and 72% respectively [19]), it is likely that AG1024 would have a similar toxic effect on these three human schistosome species.

In conclusion, our results show that simultaneous inhibition of the functional activity of SmIRs and SmVKRs using a single chemical compound can lead *in vitro* to the death of both immature and adult stages, which is an attractive feature for an alternative drug to PZQ (whose action on immature worms is defective). Further work is needed to evaluate the potential of AG1024 to kill parasites in infected animals, but these data place this drug already as a good hit for the design of more specific anti-kinase drugs applicable to anti-schistosome chemotherapy.
